# Raman cell sorting for single-cell research

**DOI:** 10.3389/fbioe.2024.1389143

**Published:** 2024-05-20

**Authors:** Xusheng Tang, Qingyi Wu, Lindong Shang, Kunxiang Liu, Yan Ge, Peng Liang, Bei Li

**Affiliations:** ^1^ Key Laboratory of Optical System Advanced Manufacturing Technology, Chinese Academy of Sciences, State Key Laboratory of Applied Optics, Changchun Institute of Optics, Fine Mechanics and Physics, Chinese Academy of Sciences, Changchun, China; ^2^ University of Chinese Academy of Sciences, Beijing, China; ^3^ Hooke Instruments Ltd., Changchun, China

**Keywords:** single-cell research, Raman spectroscopy, Raman cell sorting, Raman signal enhancement, genomics analysis, gene sequencing

## Abstract

Cells constitute the fundamental units of living organisms. Investigating individual differences at the single-cell level facilitates an understanding of cell differentiation, development, gene expression, and cellular characteristics, unveiling the underlying laws governing life activities in depth. In recent years, the integration of single-cell manipulation and recognition technologies into detection and sorting systems has emerged as a powerful tool for advancing single-cell research. Raman cell sorting technology has garnered attention owing to its non-labeling, non-destructive detection features and the capability to analyze samples containing water. In addition, this technology can provide live cells for subsequent genomics analysis and gene sequencing. This paper emphasizes the importance of single-cell research, describes the single-cell research methods that currently exist, including single-cell manipulation and single-cell identification techniques, and highlights the advantages of Raman spectroscopy in the field of single-cell analysis by comparing it with the fluorescence-activated cell sorting (FACS) technique. It describes various existing Raman cell sorting techniques and introduces their respective advantages and disadvantages. The above techniques were compared and analyzed, considering a variety of factors. The current bottlenecks include weak single-cell spontaneous Raman signals and the requirement for a prolonged total cell exposure time, significantly constraining Raman cell sorting technology’s detection speed, efficiency, and throughput. This paper provides an overview of current methods for enhancing weak spontaneous Raman signals and their associated advantages and disadvantages. Finally, the paper outlines the detailed information related to the Raman cell sorting technology mentioned in this paper and discusses the development trends and direction of Raman cell sorting.

## 1 Introduction

A cell is the fundamental functional unit of life on earth, and the traditional study of cell populations often ignores the uniqueness of single cells (Eldar and Elowitz, 2010; Spiller et al., 2010). In cell populations, even among different single cells with identical genomic information, there is remarkable phenotypic divergence, termed “cellular functional heterogeneity.” This cellular heterogeneity emerges as a significant feature of cell populations (Ocasio et al., 2019; Mermans et al., 2023). Investigating the mechanisms at the single-cell level contributes to our understanding of cell differentiation and development (Lo Celso et al., 2009; Sun et al., 2021), gene expression characteristics, and cellular features (Cai et al., 2006; Zenobi, 2013). In addition, research at the single-cell level holds substantial significance for cancer treatment (Baslan and Hicks, 2017) and contributes to the progress of bioenergy development (Chattopadhyay and Maiti, 2021; Liu et al., 2021; Li et al., 2022).

**FIGURE 1:**
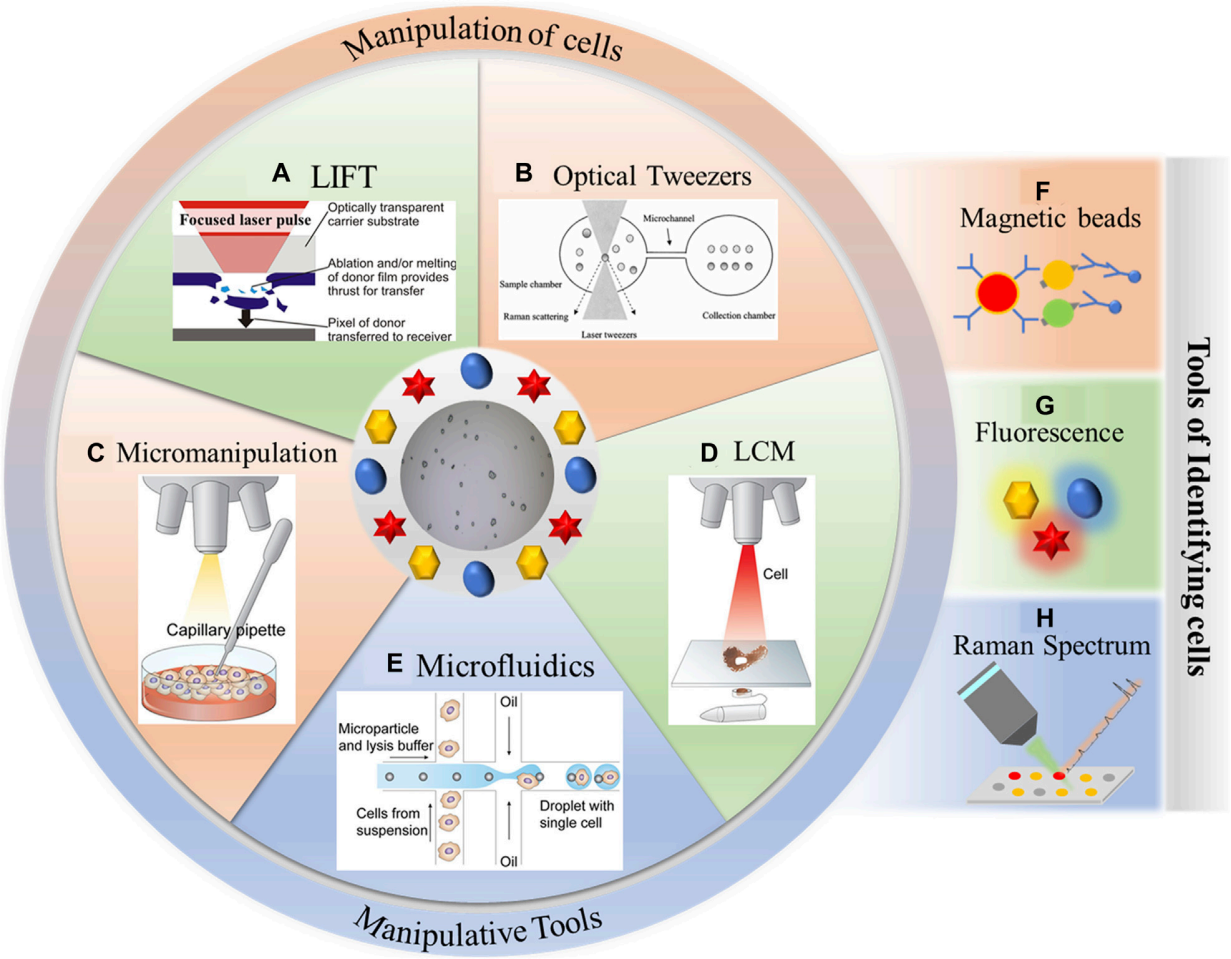
Single-cell manipulation and single-cell recognition techniques. **(A)** Laser-induced forward transfer, reproduced from Banks et al. (2009). **(B)** Optical tweezers, reproduced from Xie et al. (2005a). **(C)** Micromanipulation, reproduced from Hwang et al. (2018). **(D)** Laser capture microdissection, reproduced from Hwang et al. (2018). **(E)** Microfluidics, reproduced from Hwang et al. (2018). **(F)** Magnetic beads. **(G)** Fluorescence. **(H)** Raman spectrum.

In recent years, single-cell manipulation and single-cell recognition techniques have gradually become powerful tools for single-cell research. As shown in Figure 1, common single-cell manipulation techniques encompass micromanipulation (Fröhlich and König, 2000), laser capture microdissection (LCM) (Civita et al., 2019), microfluidics (Niculescu et al., 2021), laser-induced forward transfer (LIFT) (Serra and Piqué, 2019; Liang et al., 2022), and optical tweezers (Favre-Bulle et al., 2017; Blázquez-Castro, 2019). These techniques often necessitate a combination of recognition methods like fluorescence (Adan et al., 2017), magnetic beads (Surendran et al., 2021), and the Raman spectrum (Wagner, 2009; da Costa et al., 2019) to effectively realize the identification and sorting of single cells.

Single-cell analysis has extensively relied on optical technologies. The 20th century witnessed the emergence of new techniques, such as flow cytometry and Raman spectroscopy, which opened up novel avenues for single-cell analysis (Laerum and Farsund, 1981; Puppels et al., 1990). Optical techniques, including flow cytometry and Raman spectroscopy, play crucial roles in single-cell identification and analysis methods. The integration of these methods with single-cell sorting techniques now constitutes one of the prevailing systems for single-cell detection and sorting.

**FIGURE 2:**
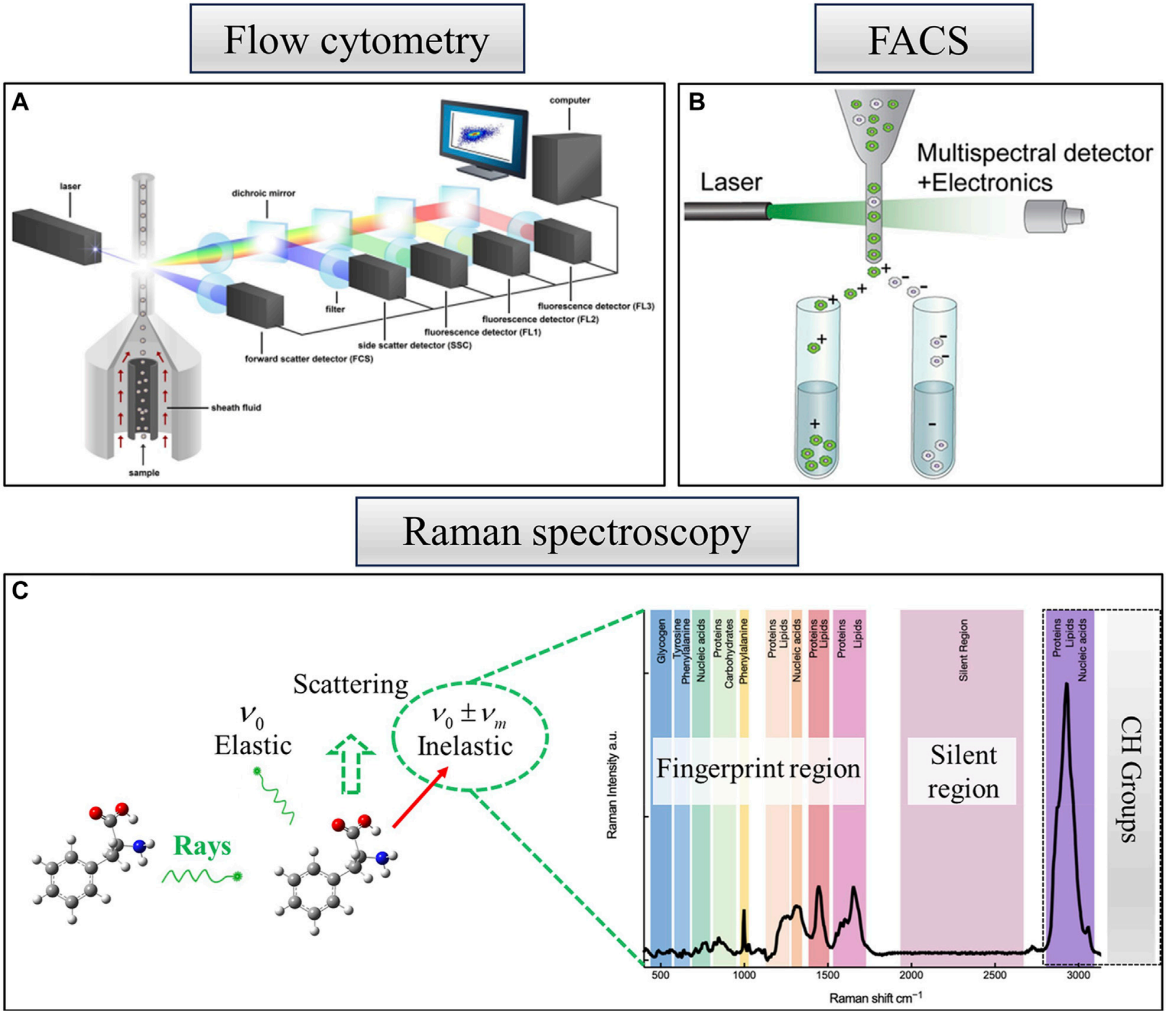
Optical techniques of single-cell identification and analysis methods. **(A)** Schematic overview of a flow cytometry analysis. Reproduced from Rubbens and Props (2021). **(B)** FACS for the identification and sorting of cells—a derivative of flow cytometry, reproduced from Hwang et al. (2018). **(C)** Raman spectroscopy—a single cell demonstrating various bands representative of cellular constituents, reproduced from Xu et al. (2021).

Microbial flow cytometry boasts a rich history, with its initial application dating back to the late 1970s (Paau et al., 1977), focusing on the examination of physiological properties within individual cultures (Paau et al., 1977; Hutter and Eipel, 1979). This technique serves not only for cell counting but also for an extensive array of studies delving into the extraction of biological information through single-cell data. High-throughput detection of properties such as cell size, intracellular complexity, and macromolecular composition is feasible (Sieracki et al., 1999; Hatzenpichler et al., 2020; Singh and Barnard, 2021). The measurement of these phenotypic properties relies on the cell labeling technique used (Müller and Nebe-von-Caron, 2010; Hatzenpichler et al., 2020; Rubbens and Props, 2021). The technique also furnishes an optical description of individual cells based on scattered light or fluorescence signals. Figure 2 provides a fundamental overview of flow cytometry analysis, wherein suspended particles are meticulously aligned one by one through hydrodynamic focusing, followed by their detection using one or more lasers (Rubbens and Props, 2021).

As shown in Figure 2B, traditional fluorescence-activated cell sorting (FACS) can be considered a derivative of flow cytometry and stands out as a robust method for the identification and sorting of cells. The FACS technique uses a laser beam to excite a single row of flowing cells, capturing their fluorescence information through a detector. Subsequently, a differently charged liquid is ejected through a piezoelectric crystal to envelop each cell. When the charged liquid encapsulating the cells traverses an electric field, it undergoes deflection toward various receiving devices, accomplishing the sorting of cells. Although flow cytometric sorting is highly rapid, it necessitates fluorescent labeling of the sample, potentially leading to sample damage (Adan et al., 2017; Hwang et al., 2018). FACS demands a substantial quantity of samples, exhibits low recovery rates, and incurs significant costs.

Fluorescent probes commonly exhibit issues such as cytotoxicity, nonspecific binding, and interference with natural cellular functions (Jensen, 2012). Many fluorescence-based methods may disrupt natural biological processes and induce damage to cells. Additionally, these probes are not universally applicable to all cell types and molecules (Jensen, 2012). Notably, fluorescent labeling faces limitations, particularly with cells like circulating tumor cells, whose surface antigens undergo frequent changes (Yu et al., 2011; Mitra et al., 2012).

Raman spectroscopy exhibits unique advantages over traditional FACS (Bonner et al., 1972). In order to avoid the drawbacks associated with fluorescent labeling, such as interference with natural cellular functions, cellular damage, and nonspecific binding with many cell types that are difficult or impossible to label, we used Raman spectroscopy, which overcomes the drawbacks. Raman spectroscopy is a biological fingerprinting technique that can reveal molecularly intrinsic information about single cells. As shown in Figure 2C, Raman scattering, a form of non-elastic scattering with a frequency different from that of incident light (Raman and Krishnan, 1928; Høgset et al., 2020; Liu et al., 2022), is gradually becoming widely utilized in biology. The figure offers a comprehensive molecular vibrational profile that encompasses the Raman bands of key cellular constituents (e.g., proteins, nucleic acids, lipids, and carbohydrates). The Raman spectra of organisms can be classified into three distinct regions (Xu et al., 2021).

1) Fingerprint region: encompassing fundamental information about biological cells (400–1800 cm^-1^). 2) Silent region: typically not involving vibration modes generated by naturally occurring isotopes in biological molecules and may include energy bands arising from stable isotopes or triple bonds (1800–2,700 cm^−1^). 3) High-wavenumber region: primarily associated with the vibration of C-H groups from lipids and proteins (2,700–3,200 cm^−1^).

The primary advantages of applying Raman spectroscopy to biological research can be succinctly outlined as the capability to analyze samples containing water, intrinsic and label-free characterization, and non-invasive, non-destructive analysis (da Costa et al., 2019). Among these, the ability to examine water-containing samples sets Raman spectroscopy apart from other vibrational spectroscopy techniques (e.g., infrared spectroscopy). In Raman spectroscopy, the low polarization rate of water molecules minimally interferes with the sample signal, and water molecules can be easily subtracted during pre-processing. This feature is particularly beneficial for the study of living cells and organisms as it eliminates the need for laborious sample manipulation, unlike the drying preparation that may potentially alter the chemical properties of the organisms themselves (Wang et al., 2016; Xu et al., 2021). Raman spectroscopy, functioning as a label-free technique, eliminates the need for *a priori* knowledge of specific substrates, and these substrates are selectively labeled. Raman spectroscopy has the capacity to display the vibrational modes of all macromolecules in a biological sample within a single spectrum, a crucial aspect for studying individual living cells.

Raman spectroscopy stands as a crucial tool for biologists, chemists, and physicists. Single-cell Raman spectra (SCRS) typically encompass more than 1,000 Raman bands corresponding to different vibrational modes of almost all chemical components, such as nucleic acids, proteins, carbohydrates, and lipids. This comprehensive profile mirrors the complex intrinsic nature of single-cell gene expression, biosynthesis of specific compounds, cellular composition, characteristic structures, and metabolic states. Consequently, Raman cell sorting has become a widely embraced analytical method for rapid, non-destructive, and non-labeled single-cell characterization (Wagner, 2009). Raman cell sorting technology will generate suitable live cells for subsequent single-cell genomics, metagenomics, and gene sequencing, and this technology will link the phenotype and genotype of live cells (Song et al., 2017; Jing et al., 2018; Li et al., 2022; Li et al., 2023).

In recent years, Raman cell sorting has gradually become a research hot spot in the field of single-cell sorting. As an emerging cell sorting method, single-cell Raman spectroscopy is usually coupled with cell manipulation and separation techniques to construct an integrated detection and sorting system (Song et al., 2016; Mermans et al., 2023). This paper introduces various existing Raman cell sorting techniques, such as Raman-activated microfluidic sorting (RAMS) (McIlvenna et al., 2016), Raman-tweezer cell sorting (RTCS) (Lee et al., 2019), Raman-activated droplet cell sorting (RADS) (Wang et al., 2017), and Raman-activated cell ejection (RACE) (Jing et al., 2018), and describes their respective advantages and disadvantages. An in-depth comparative analysis is conducted considering factors such as the probability of cell damage, the need for labeling, individual cell sorting, and high detection accuracy.

The challenge of very weak spontaneous Raman signals is predominant in Raman cell sorting techniques, and enhancement of Raman signals can help obtain Raman spectra with high signal-to-noise ratios and improve the detection efficiency and overall throughput of the system.

This paper emphasizes the importance of single-cell research and describes the currently used single-cell research methods (single-cell manipulation and single-cell identification techniques). This paper compares the traditional fluorescence-activated cell sorting technique with Raman spectroscopy and finds that Raman spectroscopy overcomes the drawbacks of fluorescent labeling, thus making Raman cell sorting a powerful tool for single-cell research due to its label-free, non-destructive detection and the capability to analyze samples containing water. In addition, Raman cell sorting technology can provide live single cells for subsequent genomics analysis and gene sequencing. By addressing the challenges encountered in Raman cell sorting, this paper provides an overview of current methods to enhance weak spontaneous Raman signals and their associated pros and cons. Ultimately, this paper outlines the detailed information related to the Raman cell sorting techniques mentioned in this paper, giving the trends and future directions of Raman cell sorting.

## 2 Common Raman cell sorting methods and techniques

**FIGURE 3:**
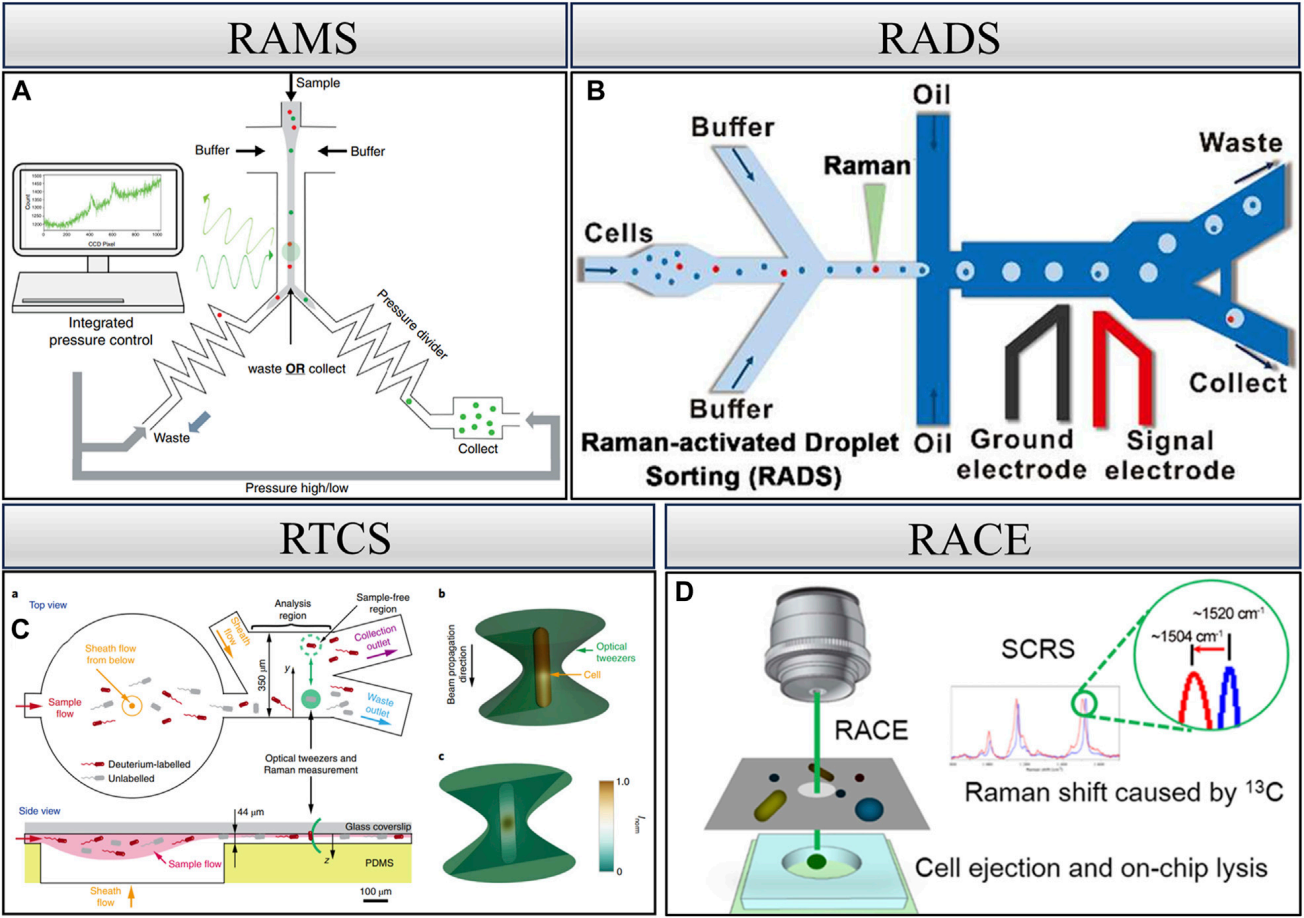
Raman single-cell sorting cytometry—systems for detecting and sorting cells based on Raman spectroscopy. **(A)** RAMS, reproduced from McIlvenna et al. (2016). **(B)** RADS, reproduced from Wang et al. (2017). **(C)** RTCS, reproduced from Lee et al. (2019). **(D)** RACE, reproduced from Jing et al. (2018).

Raman spectroscopy, based on the inelastic scattering of light, is a well-recognized, label-free, and non-destructive technique that reveals the intrinsic biochemical characteristics of cells (Butler et al., 2016) and has been utilized in the examination of single-cell phenotypes (Brauchle et al., 2016; Suhito et al., 2018; He et al., 2019; Hsu et al., 2020). In addition, after Raman detection of single cells, individual cells can still maintain their integrity and can be utilized further (Yuan et al., 2018; Lee et al., 2019). In recent years, Raman spectroscopy has been widely used by virtue of its non-labeling and non-destructive detection, which has led to the development of many Raman cell sorting techniques (Yan et al., 2021). Figure 3 depicts various techniques, including RAMS, RTCS, RADS, and RACE.

### 2.1 Raman-activated microfluidic sorting

Raman microfluidic cell sorting technology controls the flow of cells within the flow channel, and the cells remain active in the microfluidic environment. As these cells traverse the Raman excitation spot, Raman signals are generated, facilitating sample dichotomies based on the signal (Wang and Yu, 2015; McIlvenna et al., 2016). Specifically, this entails determining whether the cells belong to the target cells. As shown in Figure 3A, McIlvenna et al. developed an automated RACS system that achieves reliable synchronization of continuous Raman signal acquisition, real-time identification, and cell sorting with a sorting accuracy of 96.3%. Central to the implementation of continuous flow sorting is the use of “pressure dividers” to eliminate flow fluctuations in the inspection area. During the sorting process, Raman spectra are continuously collected. If the spectrum meets the sorting criteria, the program triggers the pump to switch output pressures and direct the flow into the collection channel. The collection channel then opens within a preset delay time before the flow is directed back to the waste outlet. Utilizing a simple hydrodynamic focusing and switching mechanism, this system eliminates the dependence on cell size, medium conductivity, and refractive index in the “capture” approach and provides a significant advantage in separating cells from complex populations (McIlvenna et al., 2016).

### 2.2 Raman-tweezer cell sorting

The optical trap generated by optical tweezers plays a dual role in manipulating target cells within the microfluidic channel. First, it restricts the flow of target cells in the channel, facilitating prolonged spectral acquisition for improved spectra. Second, it allows for the manipulation of cell movement to a specified area post-spectral acquisition. Leveraging these features, Raman optical tweezers are effective in manipulating and isolating a single cell. However, it is crucial to note that the process of isolating a single cell is very time-consuming. Previous studies have reported obtaining only a single cell using this technique within a 15-min duration (Huang et al., 2009). Subsequent research endeavors have aimed at improving the throughput of this technique. Notably, specific model microbial cells have achieved sorting throughputs ranging from 3.3 to 8.3 cells/min. However, it is only for one type of cell and does not result in only one single cell (Lee et al., 2019).

### 2.3 Raman-activated droplet sorting

The integration of droplet microfluidics with Raman cell sorting, using mutually incompatible two-phase flow to envelop cells and thereby preventing cross-contamination, represents the most efficient system for single-cell identification, sorting, and culture at present (Huys and Raes, 2018). In the RADS system, dielectrophoresis (DEP)-based droplet sorting was applied to isolate those containing target droplet cells. Notably, SCRS acquisition and DEP-based droplet sorting are controlled by multi-threading, i.e., SCRS acquisition is performed simultaneously during droplet sorting. This design further improves sorting efficiency. RADS significantly enhances the sorting throughput to over 100 cells/min (Wang et al., 2017). However, it is specific to a certain pattern of cells characterized by strong Raman signals and is not universally applicable. For instance, non-labeled *Escherichia coli* necessitates 3–5 s for the excitation of a more desirable spectrum.

### 2.4 Raman-activated cell ejection

The integration of laser-induced forward transfer technology with Raman sorting technology allows for the analysis of single-cell Raman spectra, followed by the use of laser technology for the ejection and collection of individual cells. This method measures and receives Raman spectra from single cells and enables the ejection sorting of target cells. Through subsequent single-cell whole-genome sequencing, it becomes possible to construct the relationship between single-cell genotypes and phenotypes. However, the use of laser technology poses a challenge as it can damage the cells, resulting in low genome coverage when dealing with a single cell. This technique proves highly suitable for microbial samples in complex environments like soil and sludge because there are no problems like clogging of microfluidic tubes. Additionally, it is well-suited for low-abundance samples. Despite its advantages, realizing the culture of the received single cells remains a challenging aspect of this approach (Song et al., 2017; Jing et al., 2018; Wang et al., 2020).

### 2.5 Comparison of technical parameters

This paper considers factors such as cell damage probability, the need for labeling, and single-cell sorting with high detection accuracy. Considering these factors, this paper compares the traditional FACS technique with several of the most representative Raman cell sorting techniques, as shown in Table 1.

**TABLE 1:** Comparison of FACS and several of the most representative Raman cell sorting techniques.

*Raman cell sorting techniques*	Probability of cell damage	Need for labeling	Individual cell sorting	High detection accuracy	Reference
FACS	Low	Yes	Easy	Yes	Adan et al. (2017)
RAMS	Low	No (isotope)	Hard	Yes	McIlvenna et al. (2016)
RTCS	Low	No	Yes	Yes	Huang et al. (2009)
RACE	High	No	Yes	Yes	Song et al. (2017)
RADS	Low	No (isotope)	Hard	Yes	Wang et al. (2017)

### 2.6 Raman cell sorting: genomics analysis and genome sequencing

Metagenomic sequencing and sorting are increasingly powerful tools that enable direct analysis of microbiota at the genotype level (Albertsen et al., 2013; Nielsen et al., 2014; Jing et al., 2018). However, phenotypic information on microorganisms is inevitably missed. Raman spectroscopy has proven to be a useful method to provide comprehensive phenotypic information on single cells in a non-destructive manner. Metagenomics is a culture-independent approach to microbial research. It provides a comprehensive understanding of the diversity and potential functions of microorganisms in a sample. Single-cell genomics serves as a complementary strategy to metagenomics, enabling the establishment of genetic links among DNA sequences within single cells (Swan et al., 2011; Lasken, 2012). Raman cell sorting, combined with single-cell genomics, represents a potent approach for circumventing microbial culture and revealing the microbial “dark matter.” Although fluorescence-activated cell sorting has successfully sorted single cells for subsequent single-cell genomics studies (Rinke et al., 2013), Raman cell sorting presents a valuable alternative. This method sorts cells based on SCRS, where the spectra reflect the cell’s phenotype and label-free biochemical “fingerprint” information (Huang et al., 2015). Such information can link sorted cells to their phenotypic traits and ecological functions (Song et al., 2017).

The potential of Raman cell sorting combined with single-cell genomics has been demonstrated. Song et al. used a novel RACE method to sort single bacterial cells from Red Sea water samples based on SCRS. They showed the isolation of single cells containing carotenoids from Red Sea samples based on SCRS characterization. RACE-based single-cell genomics revealed putative new functional genes associated with carotenoid and isoprenoid biosynthesis, as well as previously unknown phototrophic microorganisms, including non-culturable Cyanobacteria spp. (Song et al., 2017). Jing et al. utilized SCRS as a biochemical map, and RACE was able to link cellular phenotypes to genotypes. Mini-metagenomic sequences from RACE can be used as a reference to de-construct a near-complete genome of key functional bacteria by binning shotgun metagenomic sequencing data. This novel approach reveals the role of marine “microbial dark matter” in global carbon cycling by linking uncultured Synechococcus spp. and Pelagibacter spp. to carbon fixation and *in situ* flux activities (Jing et al., 2018).

Raman cell sorting has been applied to single-cell genome sequencing. Li et al. developed a new method combining RACS, stable isotope probing (SIP), and genome-directed cultivation (GDC). For the identification, sorting, and culture of active toluene degraders from complex microbial communities in petroleum-contaminated soil, the single cells therein were sorted and isolated using RACS. They successfully assembled the genome of *Pigmentiphaga* based on the metagenomic sequencing of 13C-DNA and genomic sequencing of sorted cells. Additionally, the genotypes and phenotypes of this degrader were directly correlated at the single-cell level (Li et al., 2023). Li et al. used 13C-labeled phenanthrene as the target and developed a new method coupling MMI−SIP and RACS. This method is designed to identify active bacterial cells that degrade phenanthrene from wastewater contaminated with polycyclic aromatic hydrocarbons (PAHs). Moreover, this approach significantly enriched active phenanthrene degraders and successfully isolated representative single cells. *Novosphingobium* was confirmed to be the active phenanthrene degrader by amplicon sequencing analysis by SIP, the 13C shift of the single cell in Raman spectra, and the 16S rRNA gene from single-cell sequencing by RACS (Li et al., 2022).

## 3 Weakly spontaneous SCRS

At present, the challenges of slow detection speed and low sorting throughput, attributed to the weak intensity of spontaneous Raman signals, severely limit the application of spontaneous Raman in the field of single-cell *in vivo* sorting. The signal from spontaneous Raman scattering is remarkably weak, with only 1 photon out of every 10^6^–10^8^ scattered photons in the sample transitioning to a Raman-scattered photon (Petry et al., 2003; Huang et al., 2010). Consequently, an extended total cell exposure time is typically necessary to acquire high signal-to-noise SCRS. Generally, the signal photon level in spontaneous Raman spectra is very low, and recording Raman spectra takes a few milliseconds to a few seconds (Zhang C. et al., 2015; Cheng and Xie, 2015). This limitation significantly hinders the detection speed and sorting throughput of Raman cell sorting systems.

## 4 Methods for Raman signal enhancement

**FIGURE 4:**
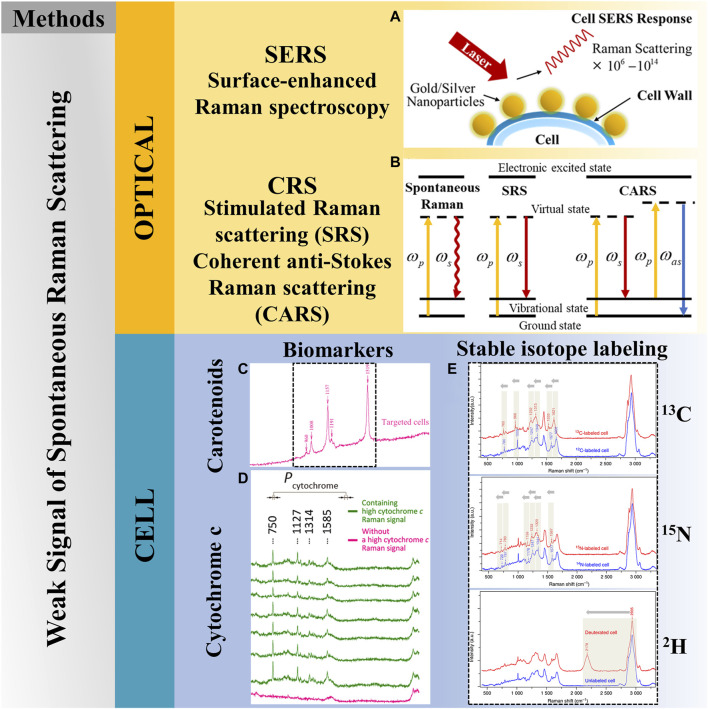
Solutions to weak signal of spontaneous Raman scattering. **(A)** SERS, reproduced from Chrimes et al. (2013). **(B)** Coherent Raman scattering. **(C)** Biomarkers—carotenoids, reproduced from Song et al. (2017). **(D)** Biomarkers-cytochrome c, reproduced from Lee et al. (2019). **(E)** Stable isotope labeling, reproduced from Wang et al. (2016).

As shown in Figure 4, to address the above issues, not only can improvements and optimizations in Raman technology be pursued but also research at the cellular level. Several solutions are currently available, including stimulated Raman scattering (SRS) (Cheng and Xie, 2015), coherent Raman scattering (CRS) (Cheng and Xie, 2015), surface-enhanced Raman spectroscopy (SERS) (Chrimes et al., 2013), biomarkers (Song et al., 2017; Lee et al., 2019), and stable isotope labeling (Wang et al., 2016).

### 4.1 Optimization for Raman microscopy: CRS

Optimized Raman microscopy can overcome the weak signal of spontaneous Raman scattering and significantly reduce the acquisition time of spontaneous SCRS. The CRS technique, a nonlinear Raman approach, involves spatially overlapping two light beams with frequencies w_p_ and w_s_, which are then converged on the sample through the objective lens. As a nonlinear optical process, it encompasses both coherent anti-Stokes Raman scattering (CARS) and SRS. These techniques arise from the nonlinear coherent Raman phenomenon, occurring when the frequency difference between the pump beam (w_p_) and the Stokes beam (w_s_) matches the Raman-active vibrational modes. They can achieve Raman signal enhancement in the 3rd–5th order (Folick et al., 2011; Zhang et al., 2014; Zhang et al., 2015c). Specifically, the CARS technique increases the velocity through a coherent radiation field, which makes the signal increased and highly directional (Evans et al., 2005; Cheng and Xie, 2015), and the SRS technique amplifies the signal through the presence of a local oscillator at the detection wavelength (Saar et al., 2010; Cheng and Xie, 2015).

Nevertheless, the application of both SRS and CAR entails the use of femtosecond lasers. Although this method drastically increases the number of photons interacting with a cell in a short duration, resulting in Raman signal amplification, it concurrently raises the likelihood of photodamage to the cell. This increased risk complicates the execution of experiments on living cells (Zhang et al., 2017; Suzuki et al., 2019). Compared to spontaneous Raman spectroscopy, which encompasses a wider range of vibrational spectra, the CRS technique covers a relatively small window of molecular vibrations (Song et al., 2016). The main limitation faced by the CRS technique is its narrow range of wave numbers.

#### 4.1.1 Stimulated Raman scattering microscopy for cellular phenotyping and sorting

**FIGURE 5:**
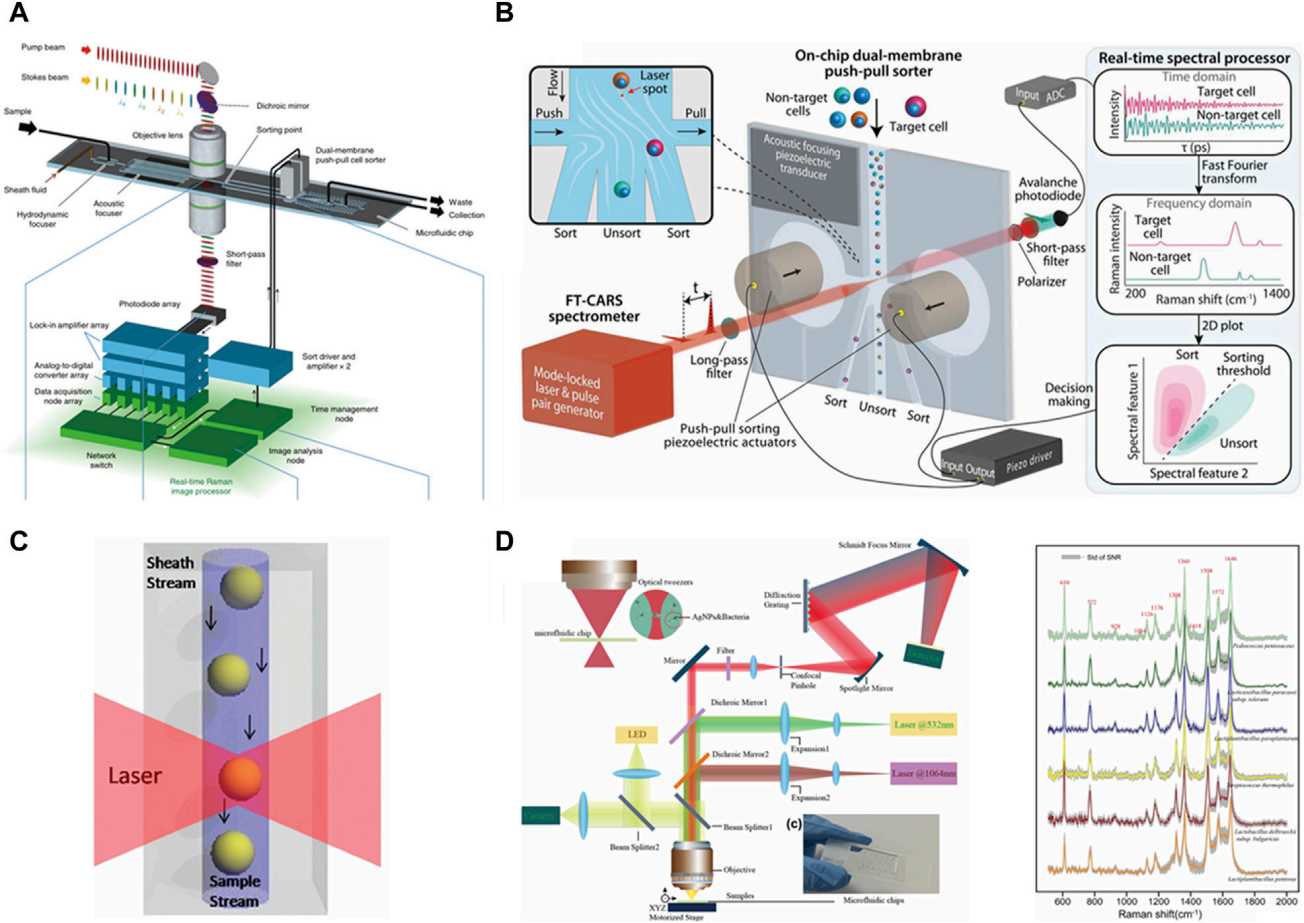
Different Raman techniques applied to Raman cell sorting. **(A)** RIACS based on SRS technology, reproduced from Nitta et al. (2020). **(B)** Raman-activated cell sorting (RACS) based on CARS technology, reproduced from Lindley et al. (2022). **(C)** SERS-based flow cytometry, reproduced from Sebba et al. (2009). **(D)** SERS technology combined with optical tweezers in a microfluidic environment, reproduced from Shang et al. (2023).

As shown in Figure 5A, Nitta et al. demonstrated Raman image-activated cell sorting technology by directly detecting chemically specific intracellular molecular vibrations for cellular phenotyping via ultrafast multicolor SRS microscopy. The system utilizes hydrodynamic focusing with acoustic focusing to converge cells into a single stream, an ultrafast multicolor SRS microscope with line-focusing geometry for continuous molecular vibration imaging of flowing cells, and an on-chip dual-membrane push–pull cell sorter for the rapid isolation of target cells from the cell stream. Specifically, the technology enables real-time sorting of individual live cells based on SRS images with a sorting throughput of up to ∼100 events per second without the need for fluorescent labeling. SRS-based Raman microscopy acquires Raman signals that are orders of magnitude faster than the signals of spontaneous Raman scattering microscopy. As a result, Raman image-activated cell sorting (RIACS) can increase sorting throughput (∼100 times higher sorting throughput) and spatial resolution by providing Raman images compared to non-imaging Raman-activated cell sorting, which can provide only one-dimensional (1D) Raman signal intensities with a moderate throughput of ∼1 eps (Nitta et al., 2020).

#### 4.1.2 Coherent anti-Stokes Raman scattering for cellular phenotyping and sorting

As shown in Figure 5B, Lindley et al. reported a coherent RACS machine utilized for cell sorting in the fingerprint region at speeds up to ≈50 eps. Fingerprint regions contain molecular backbone vibrations that typically contain richer information, and for cellular measurements, signals in the fingerprint region tend to be weaker than in the C-H region, requiring longer acquisition times. The system combines a fast-scanning Fourier-transform coherent anti-Stokes Raman scattering (FT-CARS) broadband (300–1,600 cm^−1^) spectrometer, an on-chip dual-membrane push–pull cell sorter, and a real-time spectral processor based on a field-programmable gate array (FPGA). Here, coherent Raman spectroscopy is utilized to enable Raman signal enhancement to greatly reduce the spectral acquisition time and thus increase the throughput. The acoustic-focusing piezoelectric transducer is utilized to focus the cells into a single stream, and the on-chip dual-membrane push–pull cell sorter is utilized for cell sorting. At the same time, this technique overcomes the trade-off problem of throughput versus measurement bandwidth and demonstrates broadband RACS in the fingerprint region (300–1,600 cm−1) with a record-high throughput of ≈50 cells per second (Lindley et al., 2022).

### 4.2 Optimization for Raman microscopy: SERS

The signal of Raman spectroscopy can be enhanced using SERS technology. Using surface plasmon resonance generated by nanoscale metal structures, such as metal nanoparticles, designing metal coatings with microstructures and nanostructures inside micro-orifices, the Raman spectral signal can be enhanced by 10^6^–10^14^ orders of magnitude, and a new Raman spectrum can be obtained (Chauvet et al., 2017). However, it is important to note that SERS is confined to a short range, which only has an extremely strong signal enhancement effect on chemicals within tens of nanometers close to the metal surface. In contrast, there is no significant enhancement effect on chemicals located far away from the metal surface, potentially introducing signal acquisition bias and a loss of representativeness of the Raman signal.

#### 4.2.1 SERS for cellular phenotyping and sorting

SERS exists for the rapid acquisition of SCRS in combination with flow cytometry (Watson et al., 2008; Sebba et al., 2009; Zhang et al., 2015c). As shown in Figure 5C, in SERS-based flow cytometry, it is possible to detect Raman scattering from individual tags within sub-millisecond interrogation times (Sebba et al., 2009). It can detect leukemia and lymphoma cells (MacLaughlin et al., 2013).

For rapid SERS combined with Raman cell sorting, it is important to generate more reproducible and stronger SERS signals. As shown in Figure 5D, Shang et al. combined label-free SERS and optical tweezers to construct a test platform in a microfluidic environment and performed SERS spectroscopy on six lactic acid bacteria in a microfluidic environment, verifying that the stability of the SERS spectra was improved. This technique can be used for the rapid identification of lactic acid bacteria. The peaks of each lactic acid bacterium were significantly enhanced after SERS enhancement. This study revealed that the immobilization of bacteria using optical tweezers can significantly improve the stability of SERS spectra, expanding the practical application of SERS in bacterial detection (Shang et al., 2023).

### 4.3 Cellular level: biomarkers of unlabeled compounds with strong Raman signals

Research on the production of unlabeled compounds featuring strong Raman signals, known as biomarkers, is widespread. This method involves utilizing cell-intrinsic substances that are sensitive to Raman excitation and exhibit significant enhancement in a specific region of the Raman spectrum, aiming to achieve single-cell recognition and differentiation. Carotenoids (Jehlička et al., 2014; Song et al., 2017) and cytochrome c (Pätzold et al., 2008; Lee et al., 2019) are currently commonly used biomarkers. However, it is crucial to recognize that this technique can only work for specific organisms.

#### 4.3.1 Carotenoids

Carotenoids represent a group of different chemicals with a conjugated polyene skeletal backbone. The presence of this polyene backbone enhances the Raman signal, allowing for the detection of very small amounts of carotenoids (Vítek et al., 2009; Dartnell et al., 2012). The application of Raman resonance spectroscopy to investigate natural pigments, particularly carotenoids and chlorophylls (Jehlička et al., 2014), commenced in the 1980s (Merlin, 1985), building upon the seminal work of Euler and Hellström (1932). Their research was done a few years after the discovery of the Raman effect, and the Raman spectra of carotenoids were subsequently published in 1970 (Gill et al., 1970). In 1986, the literature revealed the ability to readily detect trace amounts of carotenoids in complex substances and even in single living cells, albeit within a short timeframe (Wagner, 1986).

Carotenoids are among the most structurally diverse pigments in bacteria and are capable of generating strong resonance Raman signals (McIlvenna et al., 2016). Carotenoids are present in virtually all photosynthetic cells and have been used as intrinsic biomarkers indicative of cellular physiological function (Li et al., 2012a).

#### 4.3.2 Cytochrome c

In 1972, the resonance Raman effect of cytochrome c (Cyt c) was discovered and meticulously analyzed (Spiro and Strekas, 1972). The amplification of its Raman scattering resonance was ascribed to the electronic absorption of Cyt c in the visible spectrum (Q0 or Q1 bands) and near-ultraviolet range (B or Soret bands), corresponding to the in-plane ( $\pi \to \pi^{*}$ ) leaps of the porphyrin ring (Pätzold et al., 2008).

**FIGURE 6:**
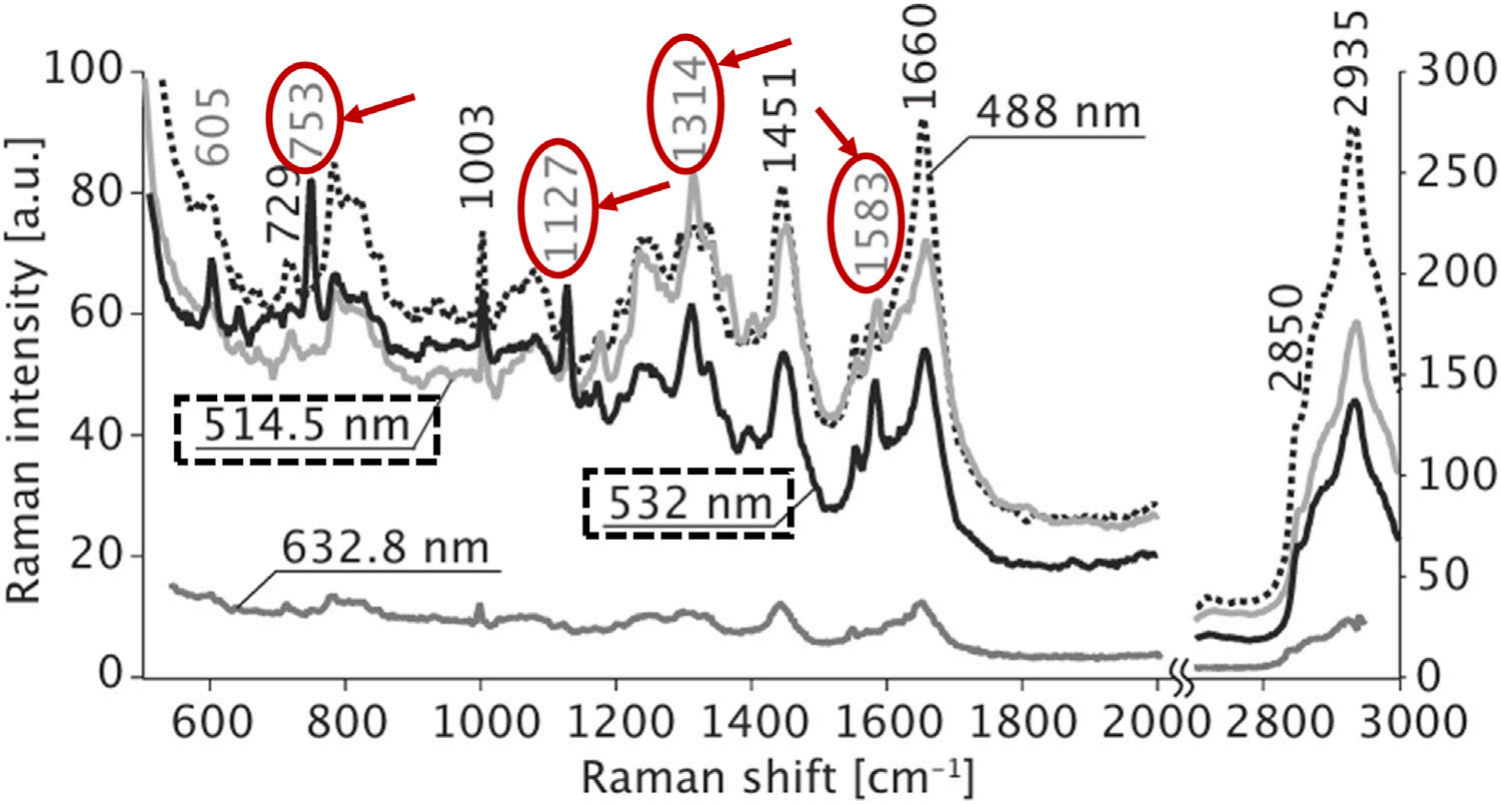
Raman spectra obtained from the cytosol of a living HeLa cell. The cells were irradiated with laser light of 488-, 514.5-, 532-, and 632.8-nm wavelengths, reproduced from Hamada et al. (2008).

Hamada et al. demonstrated the dynamic imaging of molecular distributions in unstained living cells using Raman scattering, revealing that a 532 nm excitation wavelength produced a robust Raman scattering signal. As shown in Figure 6, strong Raman peaks emerged at 753, 1,127, 1,314, and 1,583 cm in the spectra acquired with 514.5 nm and 532 nm excitation wavelengths. These peaks can be assigned to the vibrational modes of cytochrome c. Since cytochrome c contains heme proteins that absorb light from 510 nm to 550 nm, strong resonance Raman scattering is observed when irradiated in this wavelength range (Hamada et al., 2008).

### 4.4 Cellular level: stable isotope labeling (Raman redshift)

Due to its high sensitivity to minute alterations in the vibrational frequencies of chemical bonds, Raman spectroscopy can be effectively coupled with stable isotopes (SIPs). The combination of SIPs and Raman spectroscopy is sufficiently sensitive to allow for the semi-quantitative detection of ^13^C doping at the single-cell level (Li et al., 2012b).

Stable isotope labeling (SIL) involves introducing isotope-labeled nutrients into the cell culture medium. After the cells metabolize these nutrients, the molecular structure remains stable, but the corresponding Raman spectra produce a “red shift phenomenon.” This phenomenon serves not only as an indicator of functional microorganisms but also as an indicator of microbial activity (Li et al., 2019). When combined with sequencing technology, SIL enables an in-depth analysis of the metabolism and functions of single cells within complex samples. The isotopes commonly used for single-cell Raman spectroscopic characterization include ^13^C, ^15^N, and ^2^H (Wang et al., 2016).

Investigations using isotope-labeled substrates for the functional analysis of microbial communities have two significant limitations. Initially, isotope-labeled derivatives of numerous substrates of interest are prohibitively expensive and often not commercially accessible. Second, the introduction of labeled substrates modifies the composition of the natural substrate pool, potentially leading to alterations in the activities of community members (Wang et al., 2016).

## 5 Raman signal enhancement of cells in the flow state: flow Raman

In Raman cell sorting technology, the Raman assay method for cells in the flow state, when used in combination with the cell separation method, improves the sorting throughput, and the cells can be kept active in the liquid environment, which can be used to sort and provide live cells for subsequent genomics analysis and sequencing. Due to the inherently weak signals of spontaneous Raman spectroscopy, achieving high signal-to-noise Raman spectra from a single cell in microfluidic devices often requires maintaining and precisely localizing the cell in the Raman detection region. This ensures sufficient total cell exposure time for Raman signal acquisition. Cells in the flow state should be more accurately focused on the excitation spot position to prevent cells from missing or skipping Raman detection, which, in combination with the sorting section, can increase throughput. This approach also gives the cells sufficient exposure time to enhance the Raman signal, which in turn improves the detection speed. Methods to precisely localize flowing cells can enhance Raman signals and improve Raman detection speed and efficiency. This paper reviews current methods that enable precise localization and focusing of live flowing cells to the excitation spot position with the Raman detection region in a flow state with precision.

### 5.1 Precise cellular localization: optical tweezers

**FIGURE 7:**
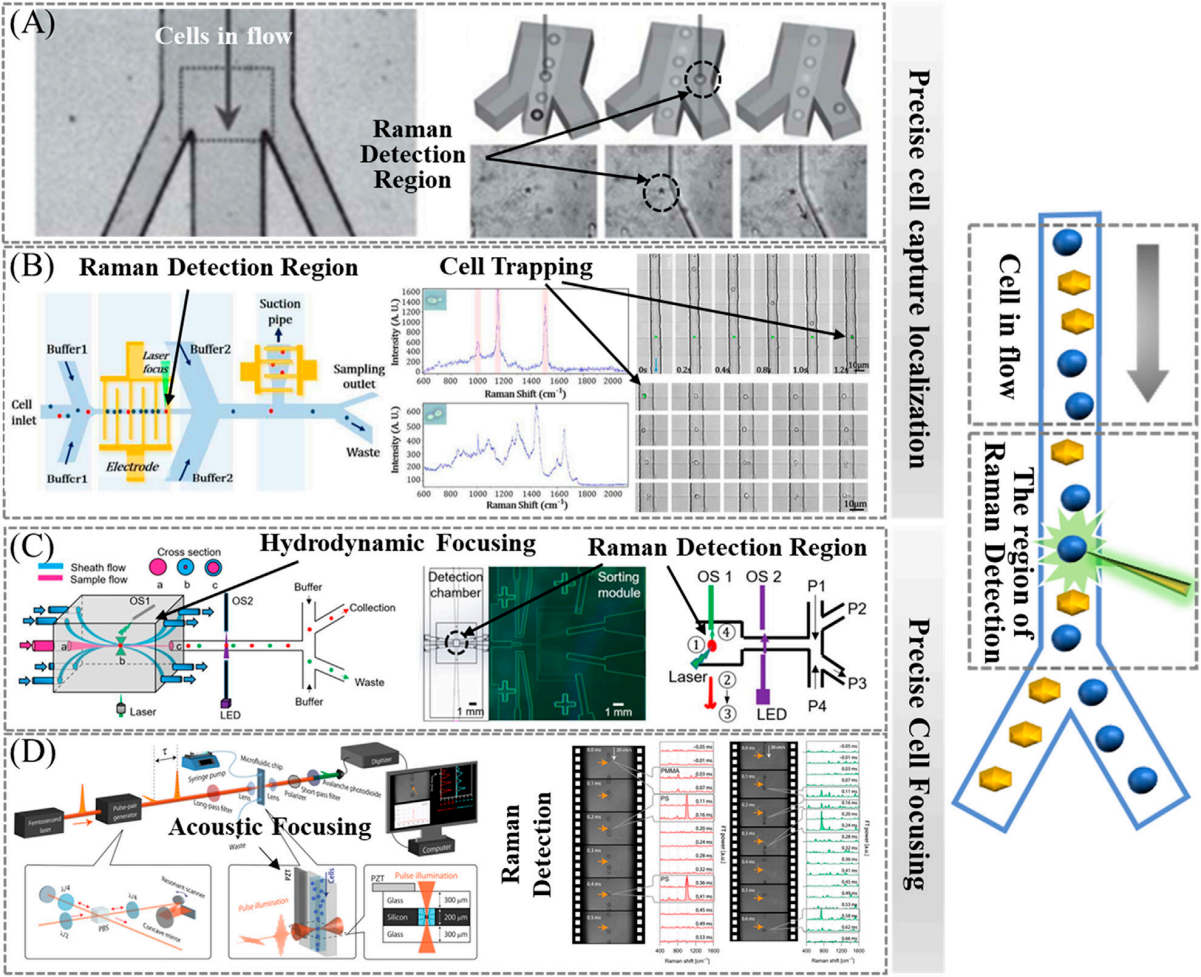
Raman signal enhancement method for cells in the flow state. **(A)** Optical-based cell capture—optical tweezers, reproduced from Lau et al. (2008). **(B)** Electric-based cell capture—DEP, reproduced from Zhang et al. (2015b). **(C)** Precise cellular localization—hydrodynamic focusing, reproduced from Lyu et al. (2020). **(D)** Precise cellular localization—acoustofluidic focusing, reproduced from Hiramatsu et al. (2019).

Laser tweezer Raman spectroscopy (LTRS) stands out as a powerful, label-free technique for manipulating and analyzing individual cells or particles in aqueous solutions. As shown in Figure 7A, Lau et al. developed an optical tweezer-based Raman cell sorting system in a flowing state, where the laser serves as an excitation source, a trapping force, and a sorting switch concurrently. This system captures cells into the Raman acquisition region, effectively extending the Raman acquisition time and prolonging the total exposure time of the cells. Subsequently, optical tweezers are used to guide cells of interest into the collection channel (Lau et al., 2008).

### 5.2 Precise cellular localization: DEP

Despite the slow capture speed and low throughput of the optical capture method known as optical tweezers, optical tweezers have been a method used to hold the position of cells during Raman detection (Xie et al., 2005b; McIlvenna et al., 2016). There is a high risk of photodamage to cells when using laser tweezers (Pilat et al., 2013). Currently, there are faster cell-trapping methods, such as DEP, which operates through electrical means. DEP can exert forces in the range of 0.1 nN–1 nN for particle manipulation, making it robust enough to capture single cells at high flow rates. Combining DEP with Raman spectroscopy facilitates label-free manipulation and cell identification, thereby contributing to an improvement in sorting throughput (Zhang et al., 2015b; Li and Anand, 2018). However, this method is based on low-conductivity, non-physiological buffers, the use of which may affect cell viability (Schroder et al., 2013).

As shown in Figure 7B, Zhang et al. developed a pioneering Raman cell sorting platform that merges positive dielectrophoresis (pDEP) with RACS. By periodically applying and turning off the pDEP field on the electrode array, the platform achieves precise capture, sorting, and delivery of single cells to the Raman detection region. Raman spectra are subsequently measured one by one. In addition, the capture time of this platform is adjustable. As a proof of concept, the platform successfully sorted carotenoid-producing and non-carotenoid-producing yeast cells. The characteristic peaks in the Raman spectra of carotenoid-producing yeast cells were markedly distinct from those of their non-carotenoid-producing counterparts, resulting in an 8-fold enrichment. This outcome serves as further evidence that the method effectively addresses the issue of weak spontaneous Raman signals. Additionally, the method exhibited notable high-throughput capabilities, capturing and characterizing at least 577 cells within a 540-s timeframe (Zhang et al., 2015b; Li and Anand, 2018).

### 5.3 Precise cellular focusing: hydrodynamic focusing

In the absence of “cell capture,” achieving precise focusing of flowing cells onto the excitation spot becomes challenging, especially in wide channels. Hydrodynamic focusing offers a solution that is independent of the physical properties of the cells and the medium, making it universally applicable to a broad spectrum of biological systems. As shown in Figure 7C, Lyu et al. developed a 3D hydrodynamically focused microfluidic system for fully automated, continuously Raman-activated cell sorting (3D-RACS). This system incorporates a 3D-printed detection chamber (1 mm^3^) integrating a PDMS-based sorting unit, optical sensors, and an in-line collection module. It is able to precisely localize cells in the detection chamber and perform Raman measurements, effectively eliminating spectral interferences from the device material. As a proof-of-concept, Raman-activated sorting of a mixture of *Chlorella vulgaris* and *E. coli* was performed with a purity of 92.0% and a throughput of 310 cells/min. This capture-free system exhibits significant potential for high throughput (Lyu et al., 2020).

### 5.4 Precise cellular focusing: acoustofluidic focusing

As shown in Figure 7D, Hiramatsu et al. used an acoustofluidic focusing microfluidic chip to tightly focus each cell in a high-speed flow, ensuring stable and efficient acquisition of Raman spectra. An acoustic standing wave, generated by a piezoelectric transducer (PZT), plays a crucial role in achieving accurate and consistent measurements in the context of a high-speed flow. This standing wave focuses cells onto a standing wave node positioned at the center of the microchannel. In this manner, all cells are tightly focused in a single stream, facilitating optical interrogation at the center of the microchannel (Hiramatsu et al., 2019).

### 5.5 Conclusion

Capturing and localizing cells precisely within the Raman detection area facilitates prolonged signal acquisition. This capability extends the total exposure time of the cells, consequently enhancing the signal-to-noise ratio of the Raman spectra. Historically, many microfluidic systems have predominantly used optical or electrical techniques, such as optical tweezers and dielectrophoresis (Zhang et al., 2015c), for accomplishing “cell capture.” Typically, Raman cell sorting techniques based on cell capture and localization methods are very common. However, the capture rate used by the above methods may have imposed a limitation on the detection throughput. In addition, capture efficiency depends on variables such as cell size, medium conductivity, and refractive index (Lyu et al., 2020).

Some methods can similarly focus cells precisely at the excitation spot position, allowing the cells to receive sufficient total cell exposure time in the Raman detection region, enhancing the spontaneous Raman signal, and improving the signal-to-noise ratio of the spectrum. Hydrodynamic focusing and acoustic focusing are common ways of precisely focusing cells in the flow state at the excitation spot position and detecting Raman spectra (flow Raman). Moreover, the integration of acoustic focusing and flow focusing is a common practice, further enhancing the precision of cell localization in the Raman detection region (Nitta et al., 2020).

Meanwhile, optical tweezers or dielectrophoresis methods often utilize hydrodynamic focusing or acoustic focusing to pre-focus flowing cells before cell capture and localization in order to capture and localize the cells in the Raman detection region more accurately, for example, by pre-focusing cells to generate a narrow stream before their capture (utilizing optical tweezers) (Lau et al., 2008). Hydrodynamic focusing typically yields elevated cell velocities, diminishing the efficiency of cell capture. In contrast, acoustofluidic focusing focuses cells at velocities conducive to effective capture (Wang et al., 2023).

## 6 Conclusion and outlooks

As shown in Table 2, this paper lists the relevant details of Raman cell sorting techniques related to this paper, such as RAMS, RTCS, RACE, RADS, RADS (DEP), RIACS, and RACS (CARS). The details include the Raman methods used, spectral ranges, capture modes, cell types, species or cell lines, whether or not resonance-enhanced, Raman analyte and throughput, etc. (Lindley et al., 2022). It can be found that the cell types or species to be sorted by the different techniques may vary, the Raman analyte may vary from cell to cell, the way to enhance the weak spontaneous Raman signal may vary, and the throughput may vary. For cellular measurements, the signal in the fingerprint region is usually weaker than the signal in the C-H region, but the fingerprint region is more informative. The fingerprint regions require longer acquisition times, and detecting larger spectral bandwidths requires longer measurement times (Lindley et al., 2022). When Raman cell sorting techniques move to a high-throughput state, the trade-off between the throughput and Raman spectral bandwidth (similar to cellular information) also needs to be de-considered.

**TABLE 2:** Detailed information on Raman cell sorting technology.

Raman cell sorting type	Raman method	Spectral region	Trapping	Cellular localization	Cell type	Species or cell line	Raman analyte	Resonance enhancement	Throughout (eps-cells/s)	Reference
RAMS	spontaneous Raman	Fingerprint	-	Hydrodynamic focusing	Cyanobacteria	Synechocystis	Carotenoids (^13^C&^12^C)	Yes	-	McIlvenna et al. (2016)
RTCS	spontaneous Raman	Fingerprint &Silent	Optical Tweezers	Hydrodynamic focusing	Bacteria	1-Escherichia coli 2-Salmonella typhimurium 3-Marinobacter adhaerens 4-Bacillus subtilis	D_2_O	-	0.055 (3.3–8.3 cells/min)	Lee et al. (2019)
RACE	spontaneous Raman	Fingerprint	-	-	Bacteria	Synechococcus spp. & Pelagibacter spp.	Carotenoids (^13^C&^12^C)	Yes	-	Jing et al. (2018)
RADS	spontaneous Raman	Fingerprint	-	Hydrodynamic focusing	Microalgae	Haematococcus pluvialis	Carotenoids(AXT)	Yes	4 (260 cells/min)	Wang et al. (2017)
RADS (DEP)	spontaneous Raman	Fingerprint	Dielectrophoresis	Hydrodynamic focusing	yeast	Saccharomyces cerevisiae	Carotenoids	Yes	1.07 (>577 cells/540 s)	Zhang et al. (2015b)
RIACS	SRS	C-H	-	Hydrodynamic focusing&Acoustofluidic focusing	Fibroblast	3T3-L1	Lipids & Protein	-	19	Nitta et al. (2020)
					Fibroblast	3T3-L1	Lipids & Protein	-	36	
					Microalgae	Chlamydomonas sp.	Lipids,Starch, Chlorophyll	-	46	
					Microalgae	Euglena gracilis	paramylon(12C&13C) &Chlorophyll	-	50	
					-	Microbeads	PS&PMMA	-	86	
					-	Microbeads	PS&PMMA			
RACS (CARS)	FTCARS	Fingerprint	-	Acoustofluidic focusing	Microalgae	1-Euglena gracilis	Paramylon	-	47	Lindley et al. (2022)
						2-Chromochloris zofingiensis	starch	-	39	
						3-Chromochloris zofingiensis	starch	-	40	
						4-Haematococcus lacustris(+)&Euglena gracilis(-)	astaxanthin	Yes	22	
					-	Microbeads	PS&PMMA	-	13	
					-	Microbeads	PS&PMMA	-	31	

Notes: ‘-’means that no such method was used or is not mentioned in the literature.

By enhancing the Raman signal and achieving faster detection with higher sorting throughput in the cell flow state, Raman cell sorting technology is expected to make significant progress in the future. In the state of cell flow, the common spontaneous Raman acquisition methods usually use a point excitation spot to achieve precise control of flowing cells by precise cell capture localization and precise cell focusing, accurately locating the cell in the Raman detection area, focusing the cell to the position of the excitation spot, and maintaining sufficient Raman acquisition time. However, these methods often require ensuring sufficient total cell exposure time in the Raman detection region to obtain spectra with good signal-to-noise ratios, which may compromise throughput. Incorporating a line-focusing strategy to detect multiple cells for SCRS in parallel (Watanabe et al., 2015; Wang et al., 2023) stands as a promising approach for the future. This process may enhance the Raman signal and improve the signal-to-noise ratio. In addition, it may enhance the speed and efficiency of detection, thus increasing the throughput. Raman cell sorting technology is capable of identifying and separating individual cells from complex cell populations in a label-free and non-invasive manner, while live cells can be obtained for subsequent genomics analysis and gene sequencing, providing a powerful means to advance the field of cell biology.
